# Will climate change increase irrigation requirements in agriculture of Central Europe? A simulation study for Northern Germany

**DOI:** 10.1186/s12302-014-0018-1

**Published:** 2014-07-22

**Authors:** Jan Riediger, Broder Breckling, Robert S Nuske, Winfried Schröder

**Affiliations:** 1 Chair of Landscape Ecology, University of Vechta, Driverstraße 22, PO Box 15 53, Vechta, 49377 Germany; 2 Northwest German Forest Research Station, Grätzelstraße 2, Göttingen, 37079 Germany

**Keywords:** European Water Framework Directive, Evapotranspiration, Soil moisture, Water availability, Water and Substance Simulation Model, Uelzen

## Abstract

**Background:**

By example of a region in Northern Germany (County of Uelzen), this study investigates whether climate change is likely to require adaption of agricultural practices such as irrigation in Central Europe. Due to sandy soils with low water retention capacity and occasional insufficient rainfall, irrigation is a basic condition for agricultural production in the county of Uelzen. Thus, in the framework of the comprehensive research cluster *Nachhaltiges Landmanagement im Norddeutschen Tiefland* (*NaLaMa-nT*), we investigated whether irrigation might need to be adapted to changing climatic conditions. To this end, results from regionalised climate change modelling were coupled with soil- and crop-specific evapotranspiration models to calculate potential amounts of irrigation to prevent crop failures. Three different runs of the climate change scenario RCP 8.5 were used for the time period until 2070.

**Results:**

The results show that the extent of probable necessary irrigation will likely increase in the future. For the scenario run with the highest temperature rise, the results suggest that the amount of ground water presently allowed to be extracted for irrigation might not be sufficient in the future to retain common agricultural pattern.

**Conclusions:**

The investigation at hand exemplifies data requirements and methods to estimate irrigation needs under climate change conditions. Restriction of ground water withdrawal by German environmental regulation may require an adaptation of crop selection and alterations in agricultural practice also in regions with comparable conditions.

## Background

Current projections of global climatic change strongly support the expectation that during the next few decades, global temperatures will continue to increase as well as spatial and temporal patterns of temperature and precipitation will be shifting [1,2]. This may have implications for many environmental processes which are influenced by thermal and soil moisture conditions. The increase in temperature for instance could lead to soil moisture deficits and a growing risk of vegetation desiccation due to increasing evapotranspiration and decreasing soil moisture [3,4]. Ecological and economic consequences for European agricultural ecosystems are expected to vary widely according to the spatial patterns of land cover, land use practise and regional climate change [5–7]. Anyway, referring for instance to Europe, according to the EU Water Framework Directive (WFD 2000/60/EC) [8], a good ecological status of surface and ground water has to be achieved. To safeguard sustainable water management, vulnerability assessments [9] and adaptation strategies based on estimates of irrigation demands are needed [10–14]. Such appraisals should be, as far as possible, spatially explicit across scales - form the local to the global [15–19] - and should regard spatial variability in terms of agricultural regions [20] and natural landscapes. As holds true for the estimation of the meteorological aspects of climate change, modelling techniques of its ecological (and economical) impacts should be used [21].

Global agriculture used about 2,600 km^3^ of water each year since the year 2000, i.e. 2% of annual precipitation over land and 17 mm of water spread evenly over the global land surface. This is a +75% increase from 1960 levels and a +400% increase from 1900 levels of irrigation. Out of the world’s croplands, 18%, i.e. about 2% of the total land surface, are irrigated and produced 40% of the world’s food. On average, the irrigated areas receive an addition of 800 mm of water each year [22]. About 70% of all water withdrawn worldwide from rivers and aquifers are used for agriculture [19]. To estimate the pressure of irrigation on the available water resources, irrigation water requirement and irrigation water withdrawal have to be assessed [23], including strategies for enhancing the water use efficiency [24]. Irrigation water requirement depends on the crop water requirement and the water naturally available to the crops (effective precipitation, soil moisture, etc.). About 2% of the global land area and 17% of the cultivated area, respectively, are irrigated. In Europe, 44% of the total water withdrawal is used for agriculture [25]. The total area equipped for irrigation, i.e. the total irrigable area in EU-27 accounts for roughly 16 million ha in 2003 and 15 million ha in 2007 on a total of 172 million ha of agricultural land; however, about 10 million ha was actually irrigated in 2007 [26]. In Germany, the area equipped for irrigation totals about 516,000 ha, and *ca*. 235,000 ha of them were irrigated [23]. In the federal state of Lower Saxony, comprising the county of Uelzen, about 3,000,000 ha, i.e. about 50% of the irrigated area of Germany is located. This irrigated area covers 11.5% of the agricultural land of Lower Saxony. In the county of Uelzen, roughly 58,000 ha are reported to be equipped for irrigation and 90% of the crop land is currently irrigated [27–31].

It is a current research task to assess whether for specific processes, regions and crops adaptation requirements will emerge under climate change conditions [32–34] to consolidate decisions on adaptation strategies to be developed and set in action. For the Northern German Lowlands, the research project ‘Sustainable Land Management in the North German Lowland’ (acronym, NaLaMa-nT, see http://www.nalama-nt.de/, funded by the Federal Ministry of Education and Research) currently evaluates agricultural implications.

**Figure 1:**
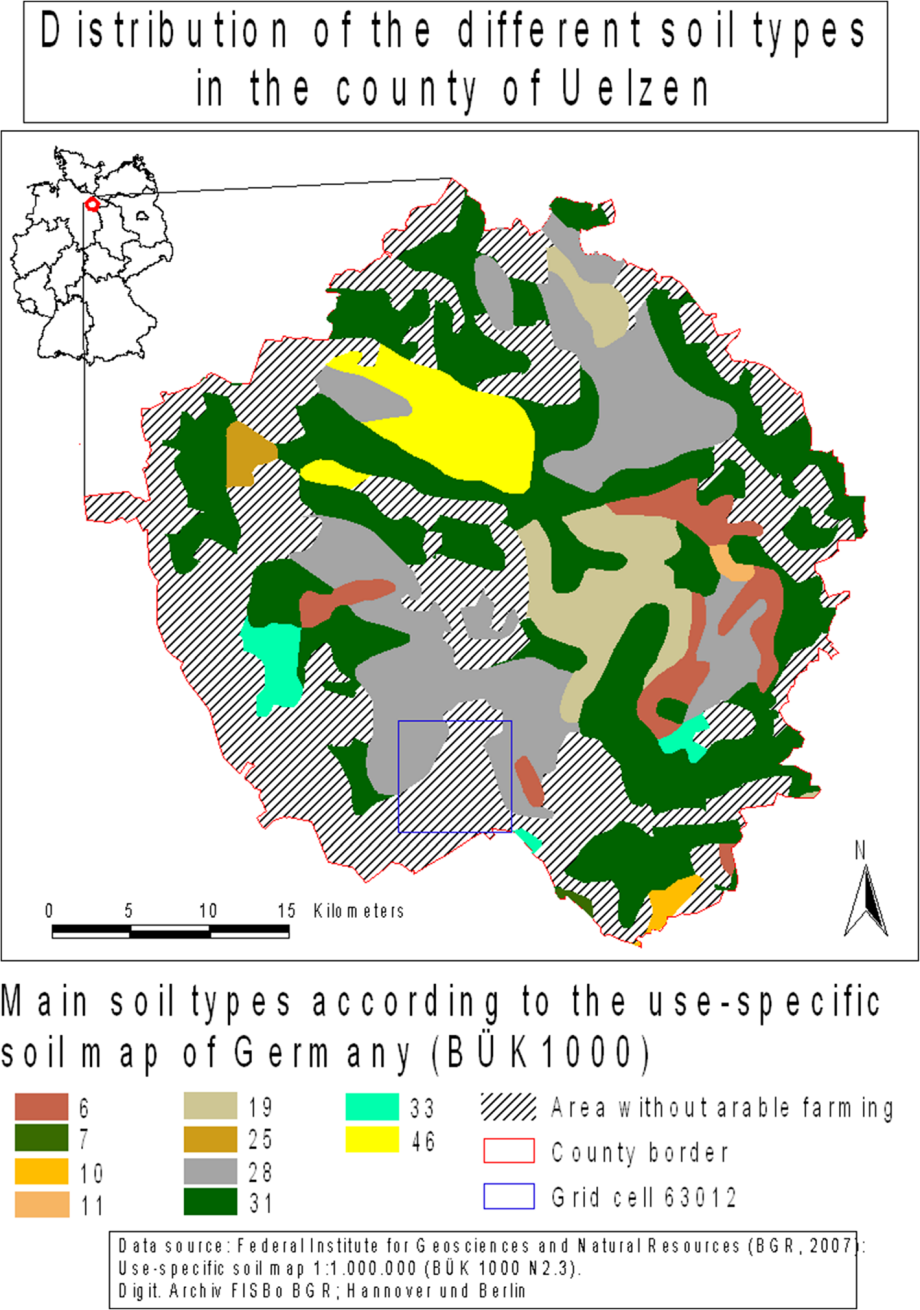
**Uelzen soil map [** 40 **].** 6 = low moor; 7 = raised moor; 10 = gleys and alluvial soils out of sand and/or loamy sand; 11 = gleys and alluvial soils out of sand, often mixed with loam and/or clay; 19 = luvisols out of loamy sand and/or loamy silt, partly stony; 25 = luvisols and pale leached soils out of slightly loamy sand; 28 = pseudogley-luvisols and pseudogley-pale leached soils out of slightly loamy sand; 31 = brown podzolic soils out of sand; 33 = podzols out of sand; 46 = lessivés, pale leached and brown soils out of fine sandy silt.

The county of Uelzen belongs to the landscape unit of the Lüneburg Heath which is located in the federal state of Lower Saxony in Northern Germany (Figure 1) [35]. Due to sub-continental climate, the county of Uelzen is characterised by comparatively low precipitation amounts (in autumn and winter 7.5 mm per month lower than the average in Lower Saxony) and relatively high daily average temperatures and slightly higher evapotranspiration amounts (in summer 3 mm per month higher compared to the whole area of Lower Saxony) [36]. Because of a low annual rainfall of approximately 600 mm per year, Uelzen is one of the counties in Germany where irrigation agriculture plays a significant role [33].

On average, 73 mm/m^2^ and year of ground water are used for irrigation in the county of Uelzen [27]. The guarantee of adequate water availability became a fundamental condition for the local economy [33]. The amount of ground water allowed to be taken for irrigation differs within the county. The local authorities have currently set ground water use for irrigation to a maximum of 79 mm/m^2^ and year with a moving average over 7 years in order to avoid depletion [27]. On average, 59.2% of the permitted amount of extracted ground water was used per year in the time period 1997 to 2004. In the low-rainfall year 2003, 125% was used. Increasing abstraction of ground water could cause negative ecological consequences, e.g. the endangerment of wetlands in the region [33]. The county of Uelzen belongs to the Elbe river basin which is the driest amongst the five largest river basins in Germany. There, the vulnerability against water stress in dry periods is currently a problem for agriculture. This scarcity is expected to increase with subsequent adverse impacts [37].

Climate change not only affects water availability but also the demand for water. If the climate in a given region gets drier and warmer, water availability will decrease and be exacerbated by increasing water demand [16]. Soil water content and therewith irrigation in agriculture is likely to be affected by higher temperature. Evapotranspiration sensitively depends on temperature regimes [38]. For temperate regions with a highly variable rainfall pattern, it is difficult to predict to which extent temperature changes will require a modification of agricultural practices. We use regional climate model data generated by STARS II [39] based on the RCP 8.5 scenario together with information on regional soil conditions [40], crop types and pattern of the current agricultural practice (by courtesy of J. Hufnagel and N. Svoboda, Leibnitz Centre for Agricultural Landscape Research (ZALF), Müncheberg, Germany) to assess whether it would be possible to continue the current cultivation pattern in Uelzen or whether adaptations and changes of the current irrigation pattern and crop management would be necessary to avoid critical conditions and crop losses - based on the assumption of a validity of the climate change scenario. Therefore, we developed the evaporation calculation model BewUe (Bewässerung (irrigation) Uelzen) to estimate the irrigation requirement using soil water content as an indicator. The presented work is part of a larger crop rotation simulation endeavour covering further Northern German regions. The computed irrigation data are essential for preparing the study of current and future water, carbon and nitrogen balances within the NaLaMa-nT project. For this purpose, the Water and Substance Simulation Model (WASMOD, [41]) was used.

## Results

**Figure 2:**
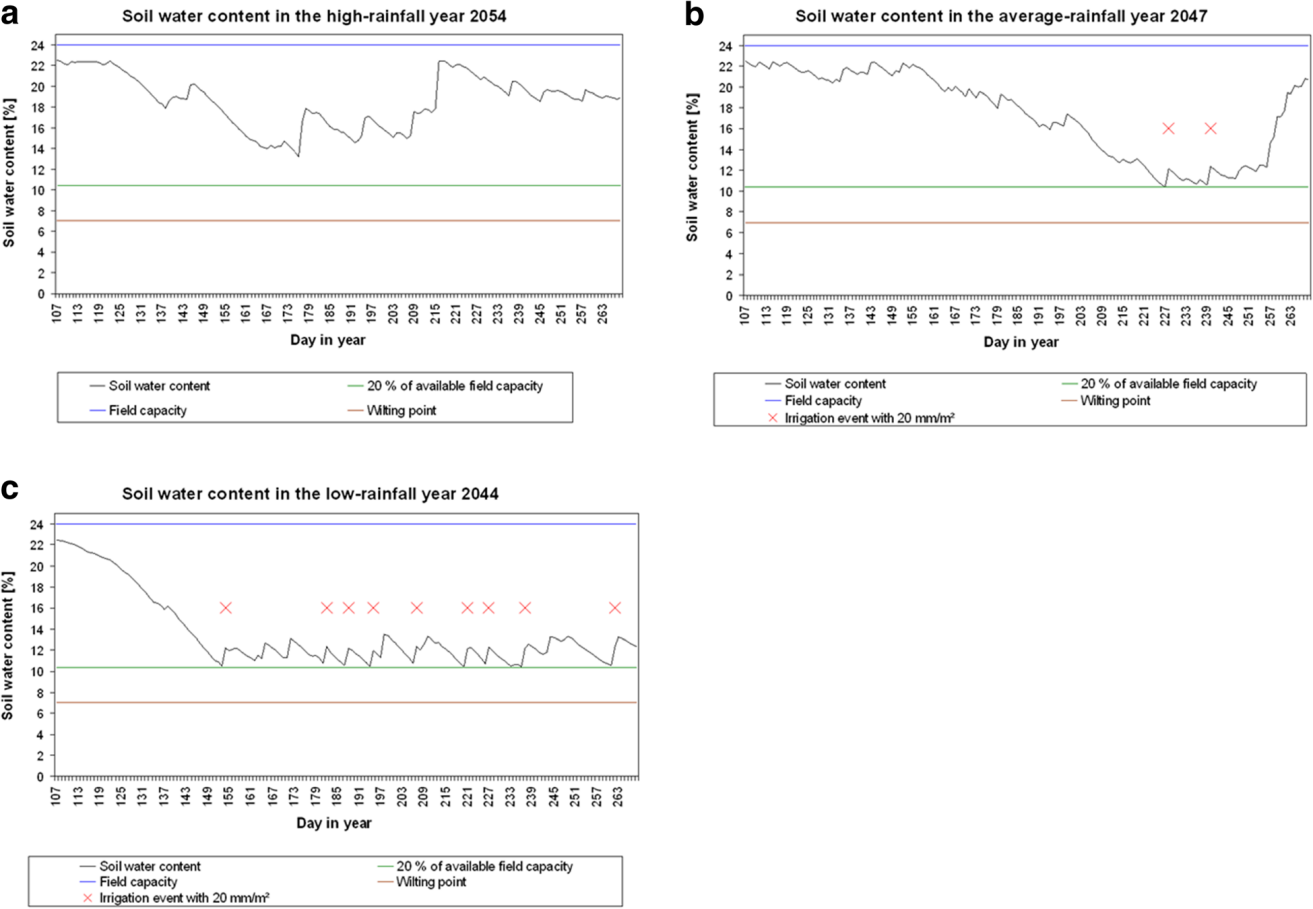
**Soil water content and computed irrigation requirements for the cultivation of sugar beet.** Soil water content as simulated by model BewUe and computed irrigation requirements for the cultivation of sugar beet. Results are shown for **(a)** the high-rainfall year 2054, **(b)** the average-rainfall year 2047 and **(c)** the low-rainfall year 2044 of the Tmax scenario run. The years were selected by the 10, 50 and 90% quantile for the annual rainfall in the time period 1951 to 2070 for scenario run Tmax of the RCP 8.5 scenario.

Irrigation requirements were calculated for all years in the reference period 1991 to 2010 and in the scenario time span 2011 to 2070. Irrigation will be applied as soon as the soil water content is lower than 20% of the available water capacity. The amount of water applied in a single irrigation event was set to 20 mm/m^2^. Typical examples illustrating the variability of soil water content and required irrigation are shown in Figure 2.

**Figure 3:**
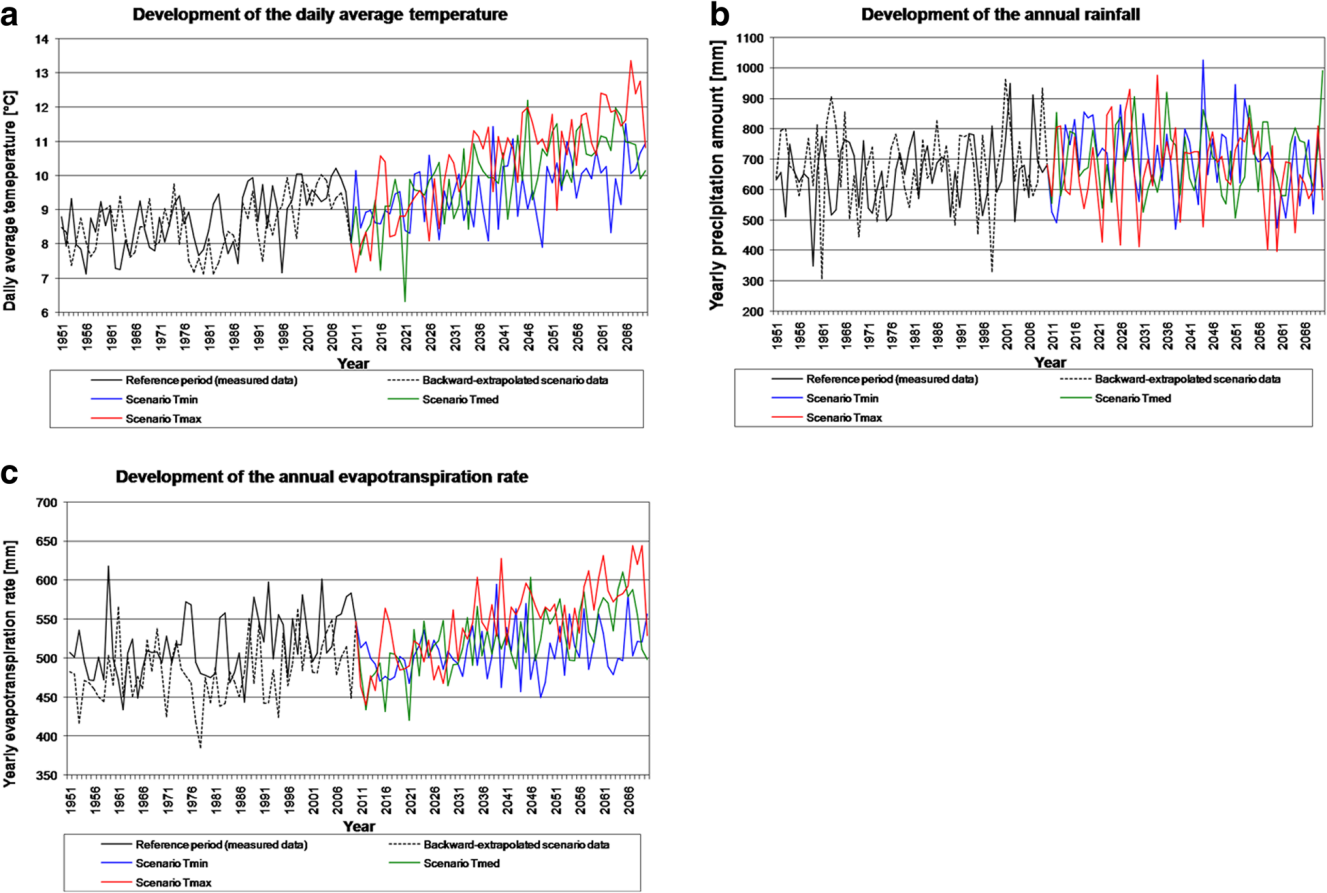
**Development of the daily average temperature, annual rainfall and evapotranspiration in Uelzen.** Development of the daily average temperature **(a)**, annual rainfall **(b)** and evapotranspiration **(c)** in the selected grid cell in the County of Uelzen (Northern Germany) during the reference period 1991 to 2010 and the different scenario runs Tmin, Tmed and Tmax (time period 2011 to 2070).

As climate conditions in terms of daily average temperature, precipitation and evapotranspiration show a high variance between different years (Figure 3) in all scenario runs; irrigation requirements also differ between the considered years (Figure 2). There was no irrigation necessary in the high-rainfall year 2054 of scenario run Tmax. The precipitation water was sufficient to grow sugar beet in this year. Two irrigation arrangements were necessary in the average-rainfall year 2047 of scenario run Tmax. With every irrigation arrangement, the soil water content increases by +2%. In the dry year 2044 of scenario run Tmax, nine irrigation arrangements were necessary to keep the soil water content sufficiently above 20% of the available field capacity. Irrigation would be still required late in the year on day 262.

**Figure 4:**
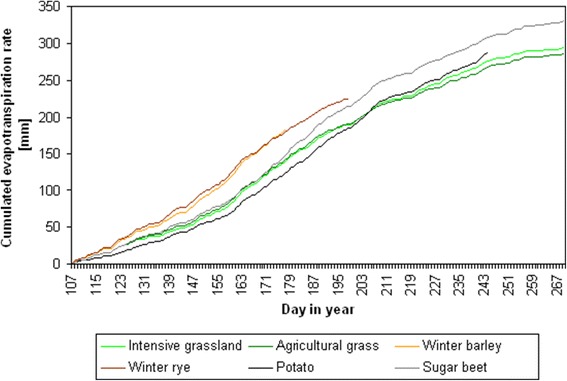
**Development of cumulated evapotranspiration rate for different grassland types and crop species during irrigation phase.** Evapotranspiration was simulated for a soil depth of 0 to 60 cm of soil type 31 (Figure 1) using WASMOD [41] in the average-rainfall year 2047 of the scenario run Tmax with two irrigation arrangements on days 228 and 240. Crop management specifications were set according to regional standards (N. Svoboda, personal communication, based on crop surveys executed at the Leibnitz Centre for Agricultural Landscape Research (ZALF) and provided to the NaLaMa-nT project data base). Curves are drawn for the irrigation relevant time span (until 3 weeks before harvest, see Methods section and Table 1).

The irrigation requirement is also related to the crop-specific evapotranspiration during the cultivation phase: crop species with a high irrigation requirement like sugar beet and potato show the highest total evapotranspiration amount (330 and 287 mm) during the irrigation phase compared to winter barley and winter rye (182 and 225 mm) with a lower irrigation requirement (Figure 4).

**Figure 5:**
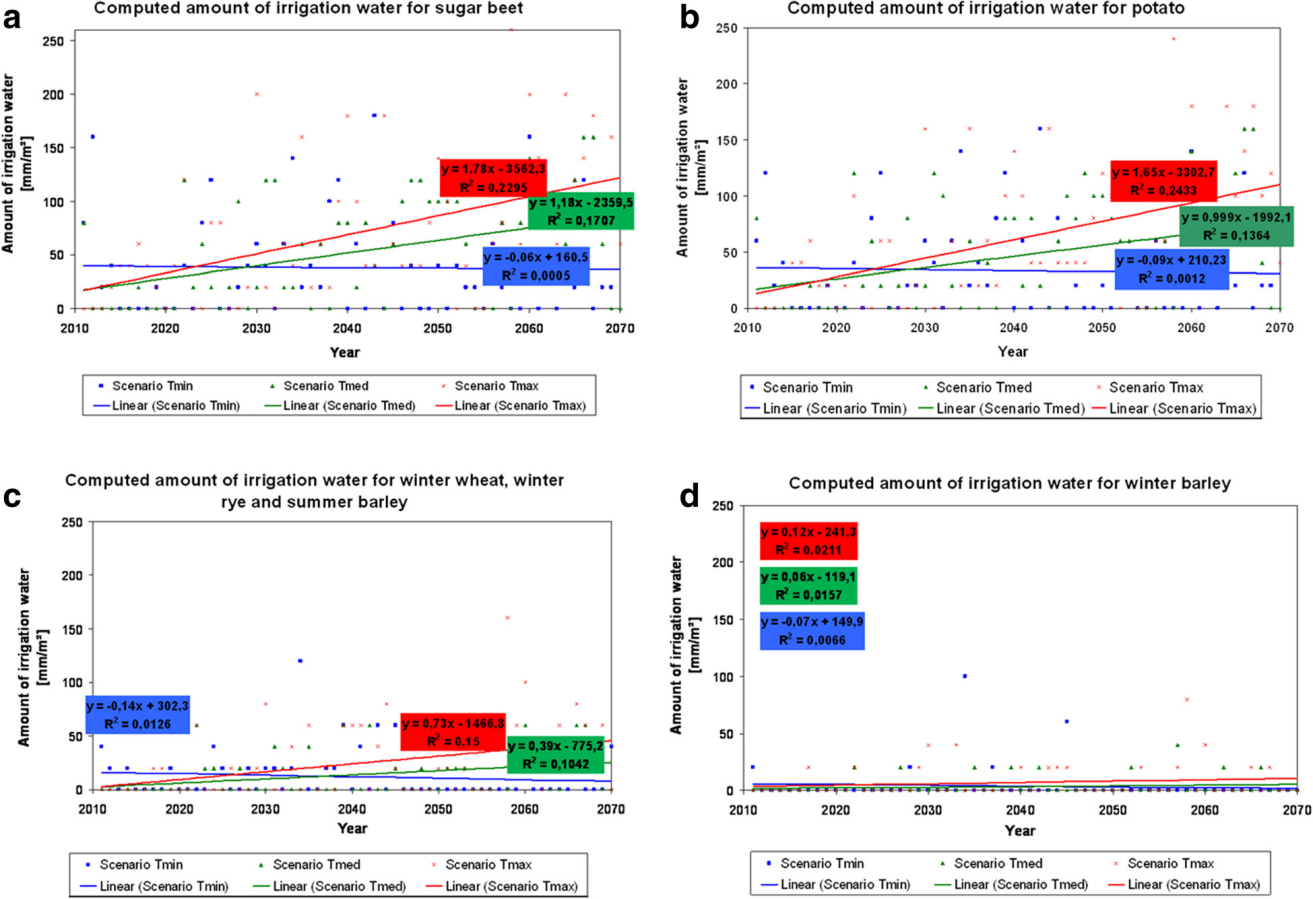
**Irrigation requirements as simulated by the model BewUe.** Results are shown for the following field crops in the different scenario runs: **(a)** sugar beet, **(b)** potato, **(c)** winter wheat, winter rye, summer barley and **(d)** winter barley. The linear regression functions and the coefficients of determination (*R* ^2^) obtained for the different crop species in the different scenario runs are also shown.

Figure 5 summarises the irrigation requirements during the considered time period. Accordingly, the irrigation requirement will increase for sugar beet, potato, winter wheat, winter rye and summer barley in the scenario runs Tmed (Kendall’s tau *b* = +0.306, +0.266 and +0.262) and Tmax (*b* = +0.374, +0.385 and 0. + 311) as average temperatures increase. The longer the crop remains on the field, the higher the irrigation requirement is and will be, respectively. For field crops, which are harvested early (e.g. winter barley), the irrigation requirement remains on a comparatively low level (*b* = −0.13 in scenario run Tmin, +0.87 in scenario run Tmed and +0.103 in scenario run Tmax). The irrigation requirement is decreasing in the scenario run Tmin for all field crops because of a decreasing evapotranspiration and a slight increase in precipitation (*b* = +0.006 for sugar beet, −0.022 for potato and −0.139 for winter wheat, winter rye and summer barley). Because of a high variance, the Kendall’s tau *b* as correlation coefficient and the coefficients of determination (*R*^2^) show low or, at most, medium values for all different field crops and scenario runs. The irrigation requirement is directly linked to the climate conditions (temperature, precipitation and evapotranspiration), which also show a high variance between the different years in all scenario runs (Figure 3).

To compute the moving average for the year 1991, the values for the six previous years 1985 to 1990 have been considered. For the year 2011, the values for the years 2005 to 2010 of the reference period 1991 to 2010 were considered. The simulated crop rotation starts with the cultivation of sugar beet in the year 1991.

**Figure 6:**
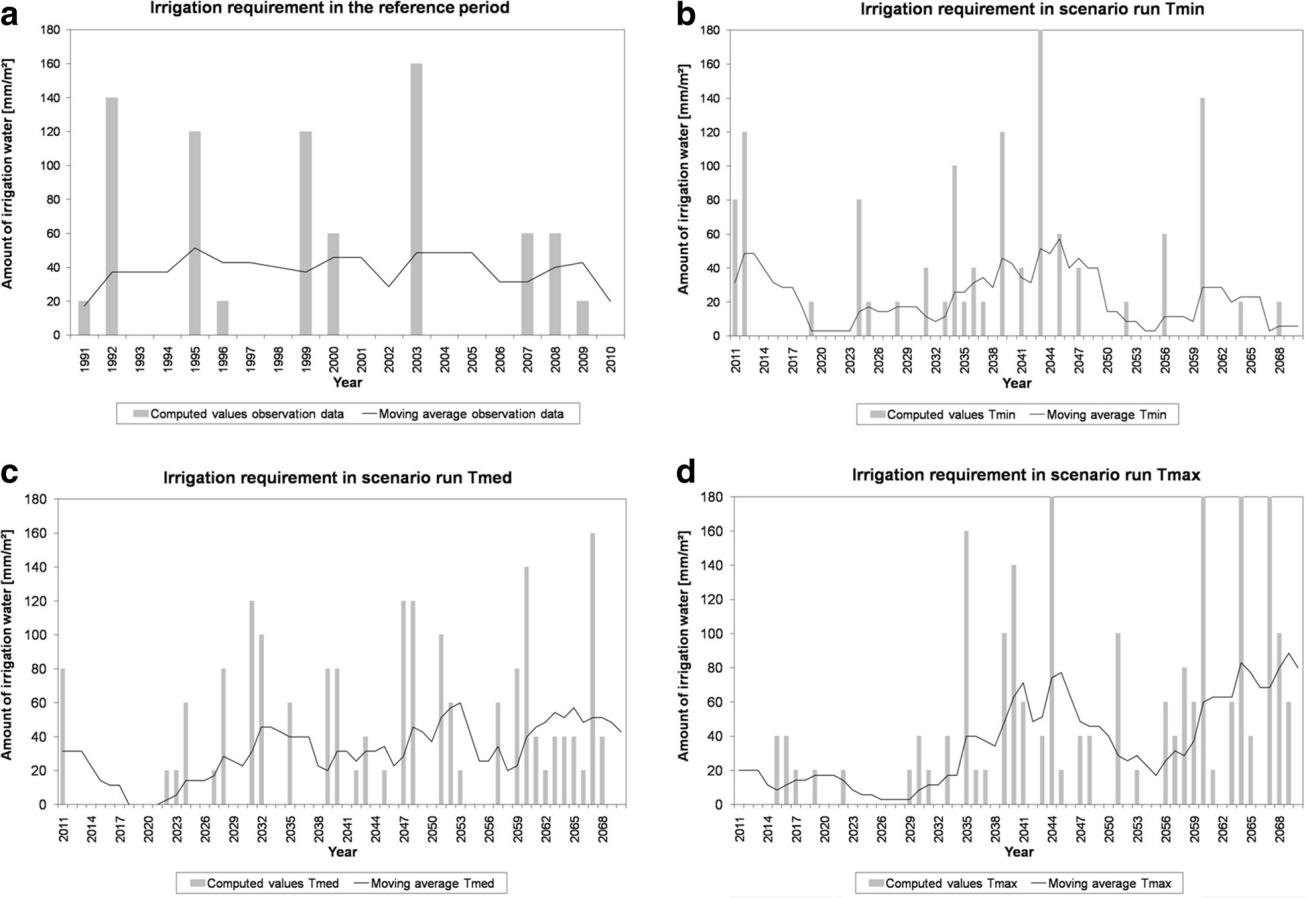
**Comparison of the moving average for a 7**-**year period and the required amount of irrigation water.** The comparison was simulated by the model BewUe for the crop rotation sugar beet-potato-winter rye-winter barley. Results are shown for **(a)** the reference period (1991 to 2010) and scenario runs Tmin **(b)**, Tmed **(c)** and Tmax **(d)** (time period 2011 to 2070).

The crop rotation sugar beet-potato-winter rye-winter barley is most common in the county of Uelzen and in particular on areas labelled as soil type 31 (brown podzolic soil out of slightly loamy, slightly silty sand and sand) (Figure 1; J. Hufnagel and N. Svoboda, ZALF, Müncheberg, Germany, personal communication). In particular, the cultivation of sugar beet and potato required large amounts of irrigation water. Figure 6 demonstrates that the irrigation requirement is likely to increase in the years after 2030 in the scenario run Tmax for this crop rotation compared to the reference period 1991 to 2010. The allowed amount of irrigation water of 79 mm/m^2^ and year in a moving average over 7 years is likely to be exhausted in several years after the year 2064. In the reference period 1991 to 2010 and in the scenario runs Tmin and Tmed, the required amount of irrigation water was always lower than 60 mm/m^2^ and year in a moving average over 7 years. In scenario run Tmed, the irrigation requirement was similar to the reference period, but in some years (2052, 2063, 2065), the moving average was slightly higher than during the reference period 1991 to 2010. In scenario run Tmin, the irrigation requirement is decreasing compared to the reference period 1991 to 2010 because of a slight increase in precipitation and a decreasing evapotranspiration.

## Discussion

The amount of water extracted for irrigation depends on climate and soil characteristics, on the political and economic boundary conditions, and on farmers’ management decisions such as crops cultivated and techniques applied, as for instance, irrigation [42]. The results of this simulation study conducted in Northern Germany (County of Uelzen) show that together with increasing temperature and evapotranspiration, the irrigation requirements are likely to increase also (Figures 3, 5, and 6). On the basis of the scenario calculations, higher irrigation levels will most likely be required in the future (except scenario run Tmin). This could exceed local availability of ground water. Implications could be, on the one hand, the endangerment of regional wetlands [33] when the ground water level should substantially decrease due to irrigation water extraction. On the other hand, crop damage or crop failure could become more common - if the land use management would not be adapted.

Both, changes in average climate conditions during the main growing season and climate variability, particularly heat waves and droughts, affect crop growth. The fitting capacity to cope with climate change depends on the crop type and ecological boundary conditions [43]. Thus, additional irrigation requirements can be avoided or reduced by a reasonably adapted choice of the cultivated field crops [33]. With an accompanying climate change, dates for sowing could be shifted - to a limited extent - to earlier times in the year to use the natural water supply during the wet months in spring more efficiently [34]. At present, irrigation is mainly applied using long-throw sprinkler [33]. If irrigation efficiency would be improved by the application of techniques with lower evaporation losses (e.g. drip irrigation), the potential problems might be reduced. In some places the use of surface water for irrigation could also be adequately adjusted and contribute to the irrigation requirements [33].

In general, scenario data - like the results shown in this paper - go along with uncertainties and are just adequate to point out possible future trends. The uncertainties in the presented calculation approach originate amongst others from climate projection data, which are linked to uncertainties themselves. For example, the used scenario data seems to predict too low evapotranspiration amounts (Figure 3). This implies that the future irrigation requirement could be even higher than the calculated values indicate (Figures 5 and 6). The presented results in particular show how irrigation requirement in the county of Uelzen could develop in the future based on the employed scenario specifications.

The presented calculation approach can be understood as being based on conservative assumptions. In reality, irrigation arrangements are likely to be done at even higher soil water contents (e.g. at 30% to 50% of the available field capacity) and with higher amounts of irrigation water than 20 mm/m^2^ and irrigation event. This leads to the assumption that the computed irrigation levels are even lower than they actually could be in the future.

In a few exceptional years, the soil water content might be lower at starting day 107 than the default start value due to a noticeably dry winter. In these cases the irrigation requirement could be higher than the calculated values indicate. Nevertheless, it could be shown that as by 2010, assuming the worst case scenario from down-scaled global climate change models, 62% to 80% of agricultural land within a Central European region could shift towards a new agroclimatic class and 98% by 2050, respectively [44].

It is important to emphasise that irrigation requirements cannot be directly derived from climate modelling. In fact, outcomes of climate modelling need to be linked with data on soil conditions, cultivation patterns and eventually also economic considerations in order to assess future sustainability of particular crops to be grown and the resulting requirements of external water input. Calculations were done with evapotranspiration data for grassland [45] based on simulated climate data [39]. This leads to uncertainties because the evapotranspiration for grassland can differ from agricultural sites depending on plant development stage and crop species. We checked this by computing evapotranspiration with a standalone model approach (Figure 4). The WASMOD results show that the evapotranspiration for sugar beet and potato differs only to a relatively low extent compared to grassland (approximately +9% and −10% on average during the irrigation phase). The variance for winter barley and winter rye is higher compared to grassland (approximately +34% and +39% on average during the irrigation phase). As winter barley and winter rye are harvested early in the year (days 177 and 198), this affects a time period when irrigation requirement is relatively low as the soil retains water from the winter period. The calculation of crop-specific transpiration in combination with plant phenology is appropriate [46]; however, it would only gradually reduce remaining uncertainties. The current approach was based on Penman-Monteith calculations [45] for site-specific conditions.

In future research activities, it will be intended to expand the calculations to additional regions, soil types and crop rotations. This would be of high relevance since the European agriculture is characterised by high productivity [47] and accounts for 50% of the global trade food products [7].

Furthermore, the presented results for irrigation amounts obtained using the BewUe programme should be cross-checked with observation data for single field crops and different years. But such observation data are not available yet. Additionally, further climate change scenarios are of interest and should be used to improve understanding of the possible future irrigation requirement.

## Conclusions

The results show that it is reasonable to expect that regional implications of global climate change will affect evapotranspiration as an important aspect in crop cultivation. Ground water availability for irrigation allows, under the given conditions, a short-term buffering towards extremes. Based on the scenario calculations, it can be expected that the current agricultural practice in the county of Uelzen will not be directly limited by regional climatic alterations. However, in the more distant future, where climate change is on the one side to become more pronounced and more uncertain to be predicted on the other side, additional measures might be necessary to prevent higher frequencies of crop failures in some years. Changes in irrigation techniques or adaptation of crop rotation types are amongst these measures. The presented work shows how irrigation requirement can be calculated combining regional projections of climate conditions specified as long-term ‘scenario-weather’, combined with crop requirements and management, and soil conditions. The results show that it is likely that agriculture in the county of Uelzen has to be adapted to increasing irrigation requirement in the future, if conditions develop as assumed in the employed scenarios.

However, further research will give evidence whether changes either in institutional and market conditions or in climatic conditions will dominantly influence the development of agriculture illustrated by a comparable example of adaptation for maize production in Switzerland [42]. Since soil climate is expected to change significantly even in Central Europe, ‘more attention should be paid to studying the impacts of climate change on soil climate’ [48], i.e. soil temperature and hydric soil regimes.

## Methods

### Data basis

Homogenised daily climate observation and scenario data for the climate change scenario Representative Concentration Pathways (RCP) 8.5 were provided by the Potsdam Institut für Klimafolgenforschung (PIK) to the NaLaMa-nT project consortium for a 10 × 10 km grid and the time periods 1951 to 2010 [36] and 2011 to 2070 [39], respectively (see Figure 3). Observation data of the German weather service [36] were checked, homogenised and interpolated to the 10-km grid [49]. The scenario data are generated by STARS II [39] and interpolated to the same grid. Our calculations were done for one grid cell located in the County of Uelzen (Figure 1), which was selected because it has the highest representativity for the county of Uelzen. Data for this grid cell show the lowest sum of deviations for the parameters of daily mean temperature, precipitation, and global radiation compared to the county average. To assess the RCP 8.5 scenario of the STARS II model scenario, backward-extrapolated scenario data for the time period 1951 to 2010 were provided and were compared to the observation data. The RCP 8.5 scenario is the most fierce emission scenario in the recent IPCC assessment [1,50,51]. Nevertheless, the RCP 8.5 scenario is surpassed by actually observed emissions [52]. It is up to the year 2060 the most similar emission scenario to the so far widely used SRES A1B scenario [1]. The used STARS II projections (Tmin, Tmed, Tmax) were driven by different temperature gradients. For each projection, the median run - based on the change of climate water balance - of 100 model runs was selected. The three projections Tmin, Tmed and Tmax are based on results from the GCM models INM-CM4 (Institute of Numerical Mathematics, Russian Academy of Sciences, Moscow, Russia, 2009), ECHAM6 (Max Planck Institute for Meteorology, Hamburg, Germany, 2012) and ACCESS1.0 (Commonwealth Scientific and Industrial Research Organisation, Melbourne, Australia, 2013). They represent a low, median and high temperature increase for the RCP 8.5 scenario.

To calculate changes in soil humidity, we used additional data for evapotranspiration, which were available for grassland correspondent to the guidelines of the Food and Agriculture Organization of the United Nations (FAO) computed by the Penman-Monteith equation [45] but not for single field crops (homogenised data source for the time period 1951 to 2010 [36] and scenario data for the time period 2011 to 2070 [39]). The scenario input data are shown in Figure 3. We used WASMOD [41] to compare the evapotranspiration amounts of different grassland types and crop species. They are in the expected order of magnitude, considering the condition that actual plant transpiration is a highly variable process leading to large standard deviations when measured in repeated experiments [53] (Figure 4).

The daily average temperature, precipitation and evapotranspiration show a high variance within the different years for the reference period 1991 to 2010 and in all scenario runs (Figure 3). This means that e.g. the general increase in temperature in scenario run Tmax is also interrupted by several years with low daily mean temperatures. The daily mean temperature is increasing in the time period 2011 to 2070 in the scenario runs Tmed and Tmax with +1.3 and +1.7°C in the time period 2011 to 2070, respectively. In the scenario run Tmin, there is a minor increase of +0.9°C on average. The annual evapotranspiration is +5.6 and +28.4 mm per year on average higher in the scenario runs Tmed and Tmax compared to the reference period 1991 to 2010. In the scenario run Tmin, the annual evapotranspiration is decreasing with −8.7 mm per year on average compared to the reference period 1991 to 2010. Concerning the annual rainfall, there is no clear trend indicating changes compared to the current situation. The annual rainfall is 654.4 mm per year during the reference period 1991 to 2010. On average, the annual rainfall is increasing in all scenario runs with +44.7 in Tmin, +45.0 in Tmed and +14.1 mm per year in Tmax compared to the reference period 1991 to 2010. Several climate scenarios also show that the main amount of the yearly precipitation will shift from the growing season (spring and summer) towards the time outside the vegetation period (i.e. winter) [33] or that the annual precipitation will even decrease [1]. This could also influence irrigation requirement.

Figure 3 also show that the modelled scenario data seem to be lower than in reality by comparing the measured climate data and the backward-extrapolated scenario data for the reference period 1991 to 2010. Temperature (Figure 3a) and especially evapotranspiration (Figure 3c) are decreasing after 2010 compared to the measured data in the reference period. Thus, irrigation requirement - calculated with observation data for the reference period and scenario data for the time period 2011 to 2070 - could be even higher in years after 2010 than the calculated values indicate.

Soil data of the land use-specific soil map of Germany [40] were used. The county of Uelzen is dominated by sandy soil types with low amounts of silt and/or clay (Figure 1). Because of the coarse-grained texture, these soils have a low available field capacity (approximately 17% to 22% in a soil depth of 0 to 30 cm) [40]. Soils in eastern Lower Saxony show a soil water deficit in some places during the growing season [54].

The information on the current agricultural practice was provided by the Leibnitz Centre for Agricultural Landscape Research (N. Svoboda, personal communication, based on crop surveys executed at the ZALF and provided to the NaLaMa-nT project data base). The dominant crops are listed in Table 1. The most common crop rotation is sugar beet-potato-winter rye-winter barley, covering 11.9% of the agricultural area.

**Table 1:** **Crop plants grown in Uelzen with dates for harvesting and starting time of maturity**

**Crop**	**Share of agricultural area (%)**	**Harvest time (day in year)**	**Start time of maturity (day in year)**
Spring barley	9	219	198
Triticale	4	219	198
Winter barley	12	198	177
Winter rye	5	219	198
Winter wheat	14	219	198
Winter rapeseed	4	183	162
Winter rapeseed with organic fertilisation		208	187
Silage maize	5	261	240
Potato	22	265	244
Sugar beet	15	290	269

Information courtesy of N. Svoboda, Leibnitz Centre for Agricultural Landscape Research (ZALF), Müncheberg, Germany.

### Methodological approach

For the calculations we used data for soil type 31 (brown podzolic soil out of slightly loamy, slightly silty sand and sand), which is the soil type most common in the county’s agricultural area (47%) (Figure 1). Soil type 31 has a field capacity of 24% (which is equivalent to a soil water content of 240 l/m^3^), an available water capacity of 17% and a wilting point of 7% (which is equivalent to 70 l/m^3^ of stagnant water) in a soil depth of 0 to 60 cm [40]. The soil depth of 0 to 60 cm was assumed to be the rooting zone of the soil and therefore the relevant part of the soil considered in our calculations.

Soil and climate data were used in the model BewUe written by B. Breckling using the programming language SIMULA [55]. The model functionality encompasses reading input data for a number of successive years and writing results into according annual output files conditionally adding irrigation when soil water content falls beneath the given threshold, i.e. it computes irrigation requirements including the day(s) of the year on which irrigation is required for single field crops and different scenario runs (Tmin, Tmed and Tmax) of the RCP 8.5 climate scenario. Irrigation requirements depend on the soil water content. We assumed that irrigation will be applied as soon as the soil water content becomes lower than 20% of the available water capacity, equivalent to a soil water content of 10.4% (which is equivalent to a soil water content of 104 l/m^3^ including 70 l/m^3^ of stagnant water and 34 l/m^3^ = 20% of available field capacity). The amount of water applied in a single irrigation event was set to 20 mm/m^2^. The main model functional is outlined in the pseudocode given in Equation 1.

1 $$ \begin{array}{l}\mathrm{Begin}\\ {}\kern1.5em \mathrm{Soil}\ \mathrm{water}\ \mathrm{content}\ \left({\mathrm{day}}_{\mathrm{n} + 1}\right) = \kern2.5em \mathrm{soil}\ \mathrm{water}\ \mathrm{content}\ \left({\mathrm{day}}_{\mathrm{n}}\right)\\ {}\kern31em  + \mathrm{precipitation}\ \mathrm{amount}\ \left({\mathrm{day}}_{\mathrm{n}}\right)\\ {}\kern36.5em \left(\mathrm{read}\ \mathrm{from}\ \mathrm{climate}\ \mathrm{data}\right)\\ {}\kern31em  - \mathrm{evapotranspiration}\ \mathrm{amount}\ \left({\mathrm{day}}_{\mathrm{n}}\right)\\ {}\kern37em \left(\mathrm{read}\ \mathrm{from}\ \mathrm{climate}\ \mathrm{data}\right)\\ {}\kern1.5em \mathrm{If}\ \mathrm{soil}\ \mathrm{water}\ \mathrm{content}\ \left({\mathrm{day}}_{\mathrm{n}}\right) < \left(\mathrm{threshold}\right)\ \mathrm{then}\ \mathrm{soil}\ \mathrm{water}\ \mathrm{content}\ \left({\mathrm{day}}_{\mathrm{n}}\right) = \\ {}\kern28em \mathrm{soil}\ \mathrm{water}\ \mathrm{content}\ \left({\mathrm{day}}_{\mathrm{n}}\right) + \\ {}\kern28.12em \mathrm{irrigation}\ \mathrm{amount}\ \mathrm{of}\ 20\ \mathrm{mm}\ /\ {\mathrm{m}}^2\\ {}\mathrm{End}\\ {}\end{array} $$

The maximum soil water content is defined as ≤ 240 l/m^3^ (soil water content at field capacity) and the minimum soil water content as ≥ 70 l/m^3^ (amount of stagnant water).

**Figure 7:**
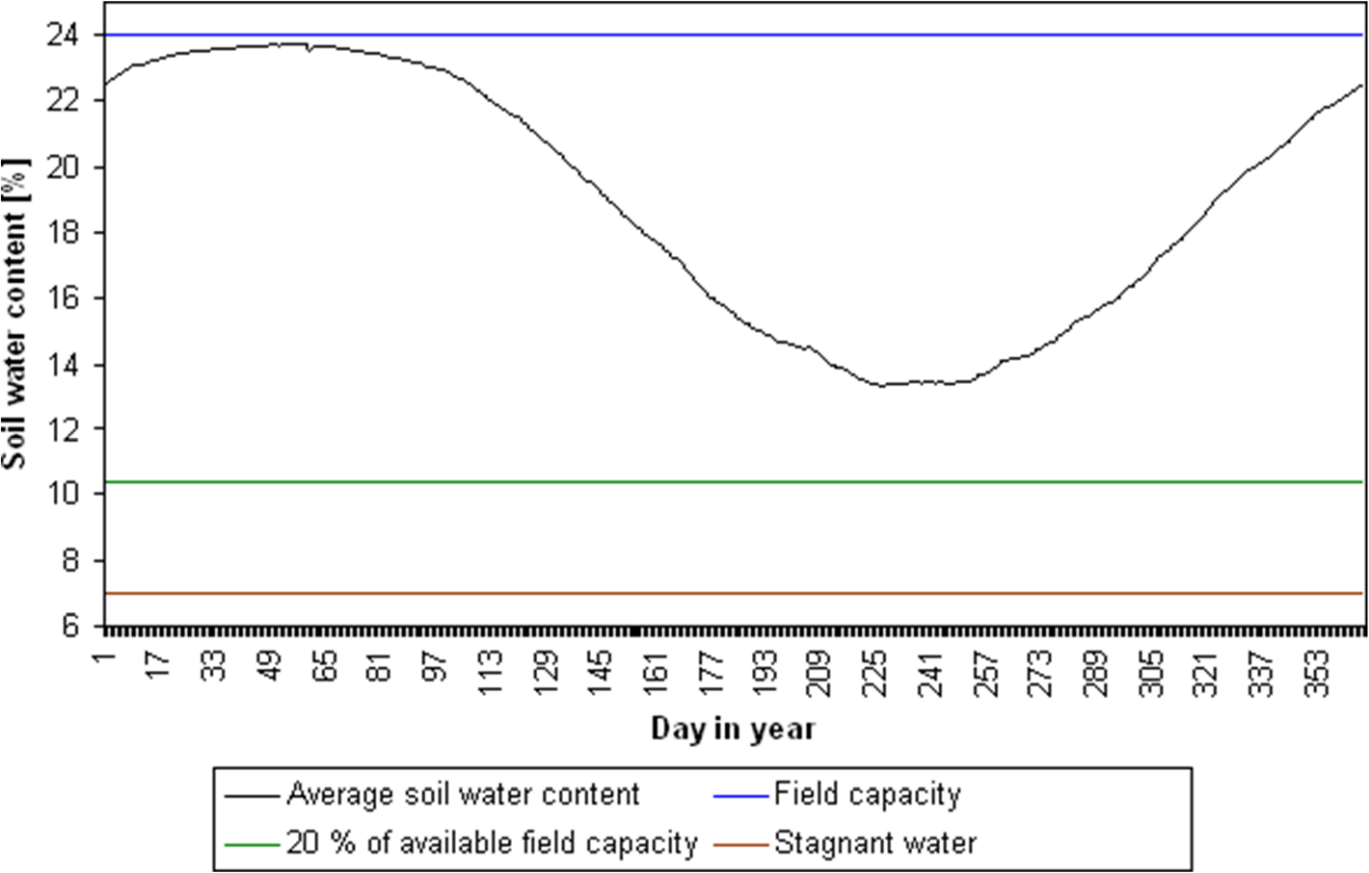
**Average soil water content for the soil type 31 of the BÜK 1000.** Average soil water content for the soil type 31 (brown podzolic soil out of slightly loamy, slightly silty sand and sand) of the BÜK 1000 [40] in a soil depth of 0 to 60 cm in the time period 1951 to 2070 (for observation data and scenario run Tmed).

In the first step, we determined the time period, in which irrigation is necessary (irrigation phase) and in which the soil water content should be computed by the use of the SIMULA model. Therefore, the soil water content was calculated for average conditions with the climate observation data for the time period 1951 to 2010 and the Tmed scenario run for the time period 2011 to 2070. For this calculation, the soil water content was set to 240 l/m^3^ on the first day (1 January 1951) assuming that the soil is fully water saturated at this time. To compute the average development of the soil water content, the daily values were averaged over the time period 1951 to 2070 (Figure 7).

The soil water content decreases noticeably after day 107 in an average year (Figure 7). In average years, irrigation should not be required before day 107. The climatic water balance gets negative from day 107 onwards because of an increasing evapotranspiration that is not compensated by precipitation. Thus, the calculation of the soil water content within the BewUe model was set to start at day 107 with an actual value of 224.9 l/m^3^. Starting time and start value are considered representative for average climate conditions in the county of Uelzen and were used to compute the irrigation requirement for all different scenario runs, years and crop species.

Irrigation was considered unnecessary during the maturation phase of the field crops. In general, maturation takes between 2 and 4 weeks depending on the yearly course of the weather (J. Hufnagel and N. Svoboda, ZALF, Müncheberg, Germany, personal communication). In our calculations, we assumed that maturation starts 3 weeks before harvesting in all years and different scenario runs. Harvest dates differ between the relevant crops (Table 1). Thus, the soil water content and irrigation requirement for e.g. sugar beet were computed from day 107 with a start value of 224.9 l/m^3^ until day 269 (i.e. start of maturation). The crop plants listed in Table 1 grow on 90% of the currently cultivated agricultural area in the county of Uelzen. In particular, the cultivation of sugar beet and potato, which are grown on 46% of the whole agricultural crop land, is linked to a high irrigation requirement [27].

Temporal trends for irrigation requirements are secured statistically by computing the Kendall’s tau *b* as correlation coefficient (Figure 5). The Mann-Kendall test is the adequate method for non-parametric tests and it is not influenced by seasonal effects.
